# Overview of surgical and anesthesia practice in sub-Saharan Africa during the 19^th^ century: the example of the people of Bunyoro

**DOI:** 10.11604/pamj.2021.40.120.32092

**Published:** 2021-10-27

**Authors:** Sylvain Diop

**Affiliations:** 1 Department of Anesthesiology and Intensive Care, Marie Lannelongue Surgical Hospital, Le Plessis-Robinson, France

**Keywords:** Caesarean delivery, surgery, Africa, traditional medicine, people of Bunyoro

## Abstract

In 1879, during a missionary expedition in the actual Uganda, the British medical student Robert Felkin witnessed a cesarean delivery surgery under general anaesthesia performed by Bunyoro´s doctors. On this occasion he saw how Bunyoro´s medicine was well elaborate in comparison with other sub-Saharan African cultures but also with occidental medicine. Through his report, Felkin brought to light the high scientific medical level of the Bunyoro´s doctors far away from the prejudice of a traditional African medicine surrounded by superstition and witchcraft.

## Commentary

**Figure 1:**
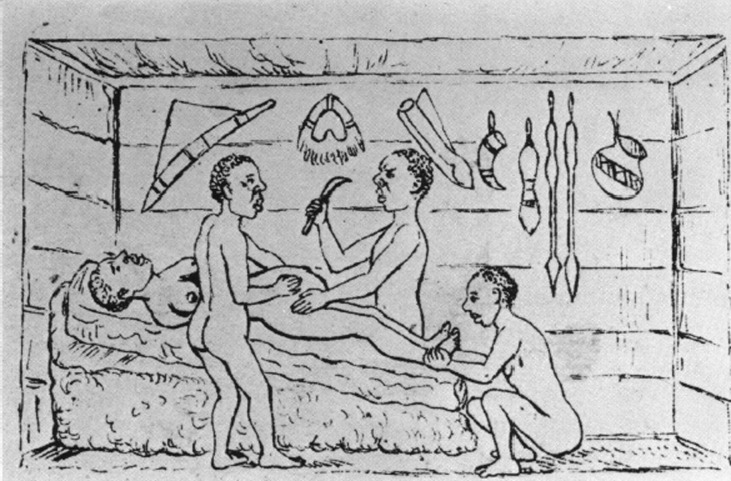
illustration from Robert Felkin showing the young woman lying on the operating table, with the surgeon’s assistant holding her ankles, as published in the Edinburgh medical journal in 1884

The following story takes place in 1879. It was reported in the *Edinburgh Medical Journal* by Robert Felkin, a British medical student, during a missionary expedition to the Bunyoro kingdom in Uganda [1]. The *Bunyoro* kingdom is a large and important African state located in the actual Western Uganda. It was founded by a Nilotic people, the Luo, in the 16^th^ century and was remarkably isolated and preserved from foreign influence until the mid 19^th^ century [2,3]. The first contact with foreigners would have been with people from Zanzibar and Egypt in 1852 [3]. Nowadays Bunyoro kingdom is still preserved with its own king and administration concomitantly with the Uganda state administration. First European contact with the people of Bunyoro, was in 1862, when two British explorers reached the northern boundaries of the kingdom [3]. They found in Bunyoro a well-structured social, political and economic system. Felkin reached the Bunyoro kingdom in 1879. As a medical student, he took a special interest in the medical knowledge of Bunyoro´s doctors. More specifically, he described in great detail a caesarean delivery that he witnessed [1]. Felkin reported that the woman was 21 years old, primigravida. First, she was anaesthetized (more accurately inebriated) with banana alcohol. She was made to lie on her back and an assistant held her ankles (Figure 1). The surgeon cleaned the woman´s abdomen with banana alcohol, and then washed his hands with the same solution. Next, he made a precise cut from the abdomen to the uterus and quickly removed the newborn baby. The umbilical cord was cut, and the uterus was emptied of the placenta and clots. Bleeding was controlled by a “white hot” metal rod. The uterus was squeezed until it contracted. No suture was performed on it. The surgeon and his assistant covered the abdominal wound with a soft grass mat and the patient was laid on her side, allowing the fluid to drain out of the abdominal cavity. Peritoneal and skin edges were tied together with iron spikes and covered with clean cloth.

Felkin observed the baby´s and the mother´s evolution for 11 days. The newborn fared well during this time. Regarding the mother, the postoperative evolution was straightforward. The iron spikes were removed from day 3 to day 6 and she showed no sign of wound or systemic infection. To understand how this story is fascinating, we must remember that, in Europe, the second part of 19^th^ century saw the establishment of two important break-throughs in the field of medical hygiene. First, the development of surgical asepsis with the work of the French microbiologist Louis Pasteur (1822-1895) and the Scottish surgeon John Lister (1827-1912) leading to the generalization of the use of carbolic acid (an antiseptic agent) on surgical wounds [4]. Second, the birth of hand-washing before surgical procedures thanks to the work of the Swiss doctor Ignace Semmelweis (1818-1865) [5]. These two discoveries represented a great revolution which led to a phenomenal decrease in post-operative infection related mortality [4]. Bunyoro´s medicine was well-developed for the time, in comparison with other sub-Saharan African cultures but also with occidental medicine [3]. Bunyoro´s physicians were familiar with the concept of surgical asepsis and hand-washing, whereas these concepts were just emerging in Europe and were welcomed with skepticism. Banana alcohol is commonly produced in western Uganda for a long time. Felkin reported that the woman was “half-intoxicated” with alcohol suggesting that Bunyoro physician used it as an anesthetic agent, to put the patient into a comatose state before surgery [1]. General and local anesthesia were widely used by physicians in some sub-Saharan people. In the Bantu region, a beer containing kaffir extract were given as pain relief to patients suffering from wounds from animal´s bite or warfare.

They also applied juices of alkaloid-containing leaves on it. Many tribes performed cataract surgery under local anesthesia, squeezing juices from alkaloid plants directly into the eyes [6]. The surgical skill itself was also astonishing and suggested a long experience of this practice [3]. Operating steps were well codified with the quick extraction of the newborn, umbilical cord clamping, surgical hemostasis with selective cauterization of uterus bleeding, fluid evacuation from the abdominal cavity, and edge sutures with specific iron spikes. As it was said by Davies in his commentary of Felkin report: “...we seem to see the evidence of analytic minds at work capable of observing over long periods and possibly capable of experimenting” [3]. There were other pieces of evidence of scientifically based medicine in the people of Bunyoro. For example, they practiced protective inoculation against endemic syphilis in children. The British physician Lambkin FJ reported in 1903 that the people of Bunyoro “inoculated infants to prevent a repetition of the disease in grown-up life” [7]. This practice was widely accepted by the people of Bunyoro and done purely for medical reasons without any religious basis [3]. Felkin´s reports were of course not welcomed when he came back to England. People doubted that a such level of knowledge and technicity was effective in Africa, notably because at this time, in Europe, African people were supposed to be “primitive” and needed to be “civilized” thus justifying the European imperialist expansion in Africa [8]. Beyond the prejudice of the African traditional medicine surrounded by witchcraft and superstition, the example of Bunyoro´s physicians illustrates that African peoples were able to develop scientific and pragmatic procedures. Felkin became a medical doctor in 1885. He continued to travel to Africa where he observed and reported medical practices among different African people. He became a distinguished doctor in the field of tropical diseases and was the advisor for the Lancet for such matters.

## References

[1] Felkin RW (1884). Notes on labour in Central Africa. Trans Edinb Obstet Soc.

[2] Dunbar A (1965). History of Bunyoro-Kitara. Oxford University Press.

[3] Davies J (1959). The development of scientific medicine in the African kingdom of Bunyoro-Kitara. Med Hist.

[4] Nakayama DK (2018). Antisepsis and asepsis and how they shaped modern surgery. Am Surg.

[5] Walter C (1976). Handwashing and Semmelweis. Ann Intern Med.

[6] Thompson EE (1965). Primitive African medical lore and witchcraft. Bull Med Libr Assoc.

[7] Lambkin F J. in D'Arcy Power and J. Keogh Murphy. A System of Syphilis, vol. ii, Oxford Univ. Press, London.

[8] Assemblée nationale Jules Ferry (2020). 1885 les fondements de la politique coloniale (28 juillet 1885).

